# Dr. Alfred P. Pierce

**Published:** 1909-08

**Authors:** 


DR. ALFRED P. PIERCE, of Bradford, Pa., died at his home in that city, August 15, 1908, aged 45 years. He graduated at the University of Buffalo, 1898. His death was due to uremia.

